# Endoscopic View of Gastroduodenal Artery Coils at the Base of Duodenal Ulcer in Case of Recurrent Massive Upper Gastrointestinal Bleed

**DOI:** 10.7759/cureus.1163

**Published:** 2017-04-13

**Authors:** Rawaa Ebrahem, Salam Kadhem, John W Frey, William Salyers

**Affiliations:** 1 Internal Medicine, University of Kansas School of Medicine-Wichita; 2 Kansas City University of Medicine and Biosciences

**Keywords:** untreated helicopter pylori, massive upper gi bleed, duodenal ulcer, arterial embolization, visible coils

## Abstract

Helicobacter pylori (H. pylori) infection is one of the major causes of bleeding peptic ulcer disease, which is associated with serious complications; therefore, the eradication of H. pylori is essential to prevent these devastating complications. Post-treatment follow-up is crucial to guarantee the eradication of the organism and may be conducted via the urea breath test, the stool antigen test, or a gastric biopsy. Acute massive upper gastrointestinal (UGI) bleeding is one of the most common complications of peptic ulcer disease. Aggressive treatment with early endoscopic hemostasis is essential for a favorable outcome. Recurrent massive nonvariceal UGI bleeding remains a challenge. Optimal management requires a multidisciplinary team of skilled endoscopists, intensivists, experienced UGI surgeons, and interventional radiologists. Endoscopy is the first-line treatment after hemodynamic stability is achieved. The role of early elective surgery or angiographic embolization in selected high-risk patients to prevent re-bleeding remains controversial.

## Introduction

A peptic ulcer is a defect in the gastric or duodenal wall that extends through the muscularis mucosa into the deeper layers of the wall. Management depends on the ulcer’s etiology, and its characteristics. Helicobacter Pylori is one of the most common causes of peptic ulcer disease, and full eradication is recommended in the following conditions: peptic ulcer disease, early mucosa-associated lymphoid tissue (MALT) type lymphoma, a family history of gastric cancer, and H. pylori infection accompanied by functional dyspepsia [1]. Full eradication of H. pylori is recommended to prevent devastating complications, such as bleeding, perforation, vascular damage, and cancer. The current treatment recommendations of the American College of Gastroenterology are the administration of proton pump inhibitor, clarithromycin, and amoxicillin or metronidazole (triple therapy) for 14 days; or bismuth with two antibiotics (quadruple therapy) for the same duration. The recommended subsequent follow-up consists of esophagogastroduodenoscopy (EGD) and a biopsy for histology, a urea breath test, or a stool antigen test to ensure full eradication of the infection and to prove healing [2-3]. In this case report, we describe an unusual finding of visible gastroduodenal arterial coils at the base of the ulcer, and the potential life-threatening complications due to poor patient compliance with the treatment and follow-up.

## Case presentation

**Figure 1:**
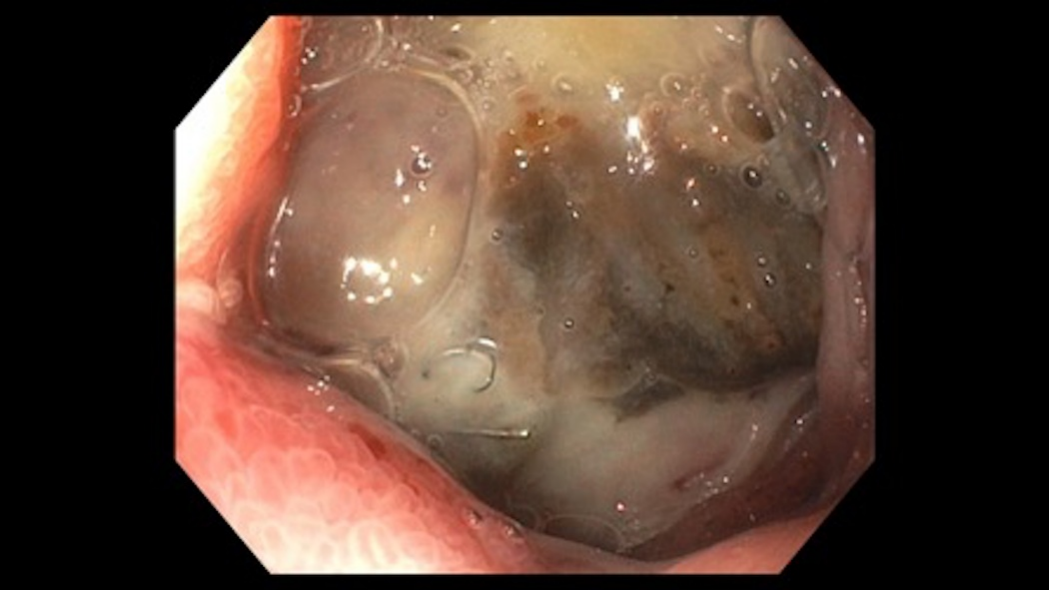
Endoscopic image shows visible arterial coiling throughout the base of a cratered duodenal ulcer.

**Figure 2:**
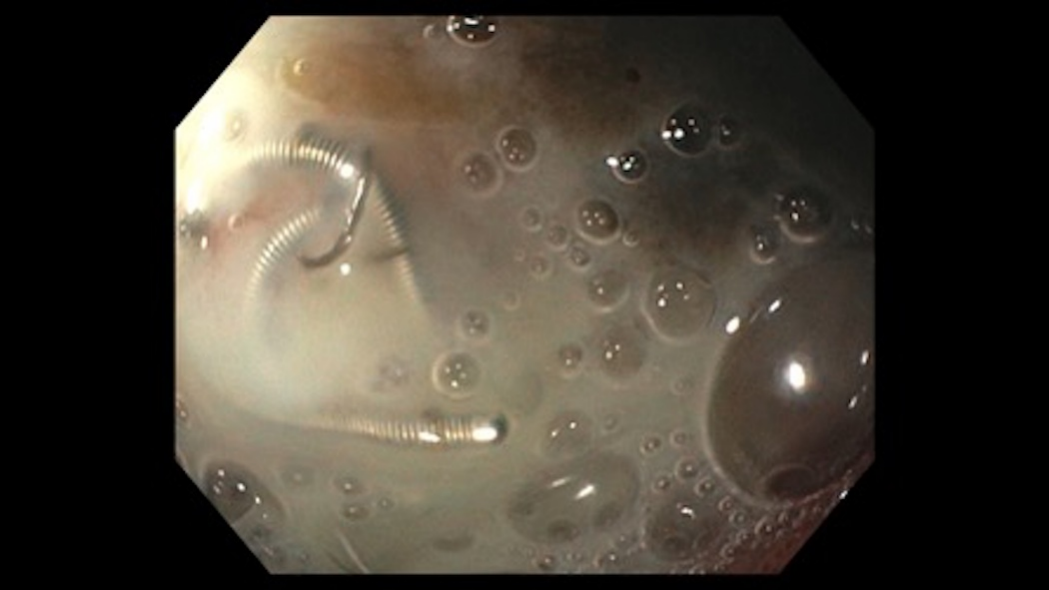
Esophagogastroduodenoscopy shows visible arterial coiling at the base of a cratered duodenal ulcer due to persistent H. pylori.

A 41-year-old female presented to the emergency department with acute epigastric pain, hematemesis, and melena. Six months earlier, the patient had undergone arterial embolization with coiling via interventional radiology to manage an actively bleeding duodenal bulb ulcer that was unresponsive to endoscopic therapy, and which was complicated by hemorrhagic shock and cardiac arrest at that time. The H. pylori serology was positive, so triple therapy (amoxicillin, clarithromycin, and omeprazole) was initiated. The patient was not compliant with medical therapy and follow-up after discharge. Upon admission, she had severe epigastric pain, tenderness, hematochezia, and hematemesis. She had been having these symptoms for two days prior to her admission. Her lab work revealed extremely low hemoglobin levels of 4.6 gm per deciliter (gm/dl), she was hemodynamically unstable, hypotensive (blood pressure 60/40 mmHg), and tachycardic (heart rate of 130 beats per minute). Fluid resuscitation, a blood transfusion, and high dose intravenous pantoprazole were initiated immediately. Following stabilization, EGD revealed a large, cratered ulcer with visible coils in the duodenal bulb (Figures 1-2). No endoscopic treatment was done at that time, there was no active bleeding, and surgery was consulted for further management.

## Discussion

This case demonstrated an unusual finding of visible gastroduodenal artery coils at the base of the ulcer, and potentially life-threatening complications due to the patient’s noncompliance with treatment and follow-up assessments. H. pylori infection is one of the most common causes of peptic ulcer disease and upper gastrointestinal (UGI) bleeding. It causes mucosal damage by eliciting a strong immune response, resulting in chemotaxis of the neutrophils, which leads to epithelial damage from cytotoxic and carcinogenic substances [4]. Antibiotic therapy is crucial for the eradication of H. pylori, which subsequently leads to the prevention of devastating complications from peptic ulcer disease, as well as reduces the risk of gastric cancer. Acute UGI bleeding is the most serious complication of peptic ulcer disease. Endoscopic treatment with local therapies like epinephrine injection, thermal therapy, and hemostatic clips are the first line for bleeding control. Despite conservative medical treatment or endoscopic intervention, severe bleeding occurs in five percent of patients. If the endoscopic therapy fails, patients may require trans-catheter arterial embolization, or super-selective catheterization with a microcatheter and embolization of the bleeding vessel with microcoils or glue if the arterial flow is not blocked by the microcatheter [5]. These options are alternatives to surgery in the case of massive gastroduodenal bleeding [6-7], with a goal of avoiding surgery, as it is associated with a mortality rate of 20-40% [8]. As a part of an effective treatment strategy, follow-up testing to confirm H. pylori eradication with EGD and biopsies for histology, a urea breath test, or a stool antigen test after a full course of antibiotics treatment is crucial to document the complete eradication of H. pylori [5]. For patients failing one course of H. pylori treatment, the recommendations are to use different combinations of antibiotics as triple therapy, or preferably, quadruple therapy. For patients failing two attempts at treatment, compliance with medications should be reinforced. A culture with antibiotic sensitivity testing can be done to guide subsequent treatment [9].

## Conclusions

Our case demonstrates the importance of completing H. pylori eradication therapy. Post-treatment follow-up via a urea breath test, a stool antigen, or EGD and biopsies are required to prove full eradication; otherwise, serious complications might be encountered which could include local erosion, perforation, and possible vascular complications including life-threatening UGI bleeding.
